# Structure and function of a CE4 deacetylase isolated from a marine environment

**DOI:** 10.1371/journal.pone.0187544

**Published:** 2017-11-06

**Authors:** Tina Rise Tuveng, Ulli Rothweiler, Gupta Udatha, Gustav Vaaje-Kolstad, Arne Smalås, Vincent G. H. Eijsink

**Affiliations:** 1 Faculty of Chemistry, Biotechnology and Food Science, Norwegian University of Life Sciences (NMBU), Ås, Norway; 2 The Norwegian Structural Biology Centre, Department of Chemistry, The Arctic University of Norway, Tromsø, Norway; Institut National de la Recherche Agronomique, FRANCE

## Abstract

Chitin, a polymer of β(1–4)-linked *N*-acetylglucosamine found in e.g. arthropods, is a valuable resource that may be used to produce chitosan and chitooligosaccharides, two compounds with considerable industrial and biomedical potential. Deacetylating enzymes may be used to tailor the properties of chitin and its derived products. Here, we describe a novel CE4 enzyme originating from a marine *Arthrobacter* species (*Ar*CE4A). Crystal structures of this novel deacetylase were determined, with and without bound chitobiose [(GlcNAc)_2_], and refined to 2.1 Å and 1.6 Å, respectively. In-depth biochemical characterization showed that *Ar*CE4A has broad substrate specificity, with higher activity against longer oligosaccharides. Mass spectrometry-based sequencing of reaction products generated from a fully acetylated pentamer showed that internal sugars are more prone to deacetylation than the ends. These enzyme properties are discussed in the light of the structure of the enzyme-ligand complex, which adds valuable information to our still rather limited knowledge on enzyme-substrate interactions in the CE4 family.

## 1. Introduction

Today there is a focus on the shift from a fossil-based economy to a greener economy based on renewable resources such as biomass. Chitin, an insoluble polymer of β-1,4 linked *N*-acetylglucosamine (GlcNAc), is considered as the second most abundant biomass on earth, and occurs in large amounts in different ecosystems, for example in the exoskeleton of crustaceans and insects. Many microorganisms can utilize chitin as an energy source and exploration of metagenomics information from chitin-rich ecosystems is thus likely to reveal enzymes with activity against chitin.

The production of chitosan [partially deacetylated chitin consisting of GlcNAc and glucosamine (GlcN)] and chitooligosaccharides (CHOS, i.e. homo- or hetero-oligosaccharides of GlcN and GlcNAc) from chitin is of considerable industrial interest. However, the extraction of chitin from e.g. shrimp shells and the subsequent production of chitosan and CHOS involves the use of harsh chemicals that are not environmentally friendly [1, 2]. Therefore, it is desirable to replace one or more of the chemical processing steps used today with enzymatic processes. The degree of polymerization (DP) and the fraction of acetylation (F_A_) are well known determinants of the physicochemical and biological properties of chitosan and CHOS. In addition, the pattern of acetylation (P_A_) is believed to have impact on the properties of chitosan and CHOS [3]. The potential applications of chitosan and CHOS are numerous (reviewed in e.g. [4] and [5]), which is in part due to their biocompatibility.

Deacetylases acting on chitin (CDAs) occur in carbohydrate esterase family 4 (CE4) of the CAZy database (www.cazy.org) [6]. CE4 enzymes are capable of removing acetyl groups in chitin, chitosan, and CHOS, thus converting GlcNAc (or A) units to GlcN (or D) units. Enzymes in the CE4 family may also act on peptidoglycan [7, 8] and acetyl xylan [9]. The use of CDAs could in principle allow tailoring of both the fraction and pattern of acetylation in chitosan and CHOS [10–12]. For example, Hamer et al. used two different deacetylases (NodB from *Rhizobium* sp. GRH2 and *Vc*CDA from *Vibrio cholerae*) to produce CHOS containing two deacetylated sugars in their non-reducing ends [12]. They could do so because NodB specifically deacetylates the non-reducing end, while *Vc*CDA specifically deacetylates the sugar next to the non-reducing end [12]. Notably, most characterized CE4 deacetylases show a broader substrate specificity [13–15], deacetylating several positions in CHOS, chitin, chitosan, and acetyl xylan.

Despite their abundance in Nature and a plethora of (potential) roles in biology and industry, available structural information for CE4 enzymes remains limited, and information on enzyme-substrate interactions is scarce. In 2014, Andrés et al. described structures of *Vc*CDA in complex with chitobiose and chitotriose. Based on this landmark study, these authors proposed that the pattern of acetylation in the products of different CE4 enzymes is determined by variable loops near the catalytic center that affect the accessibility of subsites in the binding cleft [16].

In an attempt to discover novel CDAs, we have searched a collection of bacterial genomes and metagenomes for members of the CE4 family starting from existing annotations based on the Enzyme Commission classification system [17]. Bioinformatic tools were utilized to select the most promising candidates, resulting in one candidate for cloning, expression and in-depth characterization. X-ray crystallography yielded two structures, one for the substrate free protein and one for a complex with (GlcNAc)_2_ bound in the active site. This novel CDA has an open active site (in contrast to *Vc*CDA) and the structure with substrate is the first structure of a complex for this type of deacetylase. We also elucidated the substrate specificities of this deacetylase to gain insight into its potential use for tailoring patterns of acetylation in CHOS.

## 2. Materials and methods

### 2.1 Selection of candidates

An internal collection of annotated bacterial genomes and metagenomes (~300 Mb of sequence data), supplemented with metagenomics data from an Intestinal Microbiota Project [18] and from the HOTS vertical ocean depth project, was searched for potential chitin deacetylases, i.e. enzymes annotated with E.C. number 3.5.1.41. The resulting candidate proteins (64 in total) were subjected to further bioinformatic investigations to select the most promising candidates, as described in the Results and Discussion section.

### 2.2 Cloning and protein production

Synthetic gene encoding the selected protein (without signal peptide) with an N-terminal His6-Ala-Gly-tag and sequence optimized for expression in E.coli, were ordered from GenScript (NJ, USA), amplified by PCR and cloned into the pNIC-CH [19] vector utilizing Ligation Independent Cloning [20]. The synthetic gene encoded an N-terminal His-tag and contained its normal stop codon (meaning the C-terminal His-tag encoded by this vector was not exploited). The plasmid containing the gene of interest was transformed into chemically competent BL21 Star cells by heat shock. Transformants were cultured in 2 ml LB medium supplemented with kanamycin (50 μg/ml) and a colony PCR type of method was performed to check for correct plasmid size. Cultures for strains containing plasmids with correct sizes were further cultivated by adding more LB medium and kanamycin, after which plasmids were isolated using the plasmid purification kit from Macherey-Nagel GmbH & Co (Düren, Germany), followed by sequencing of the inserted gene at GATC Biotech (Constance, Germany) using Sanger sequencing.

Protein expression was started by growing a 5 ml pre-culture (LB with 50 μg/ml kanamycin, overnight, 37 ^o^C) which was used to inoculate 0.5 L TB-medium supplemented with kanamycin (50 μg/ml) and containing 0.011% Antifoam 204 (Sigma, Steinheim, Germany), followed by incubation at 37°C in a Harbinger system (LEX-48 Bioreactor, Harbinger biotech, Markham, Canada). At OD_600_ = 0.6, the culture was induced with IPTG (final concentration 0.2 mM) and incubation was continued over night at 30°C before harvesting the cells by centrifugation. The cell pellet was resuspended in 20 ml 20 mM Tris-HCl, 150 mM NaCl, 10 mM imidazole, pH 8.0. Before sonication (28% amplitude with a pulse of 5 seconds on, 10 seconds off for 10 minutes), DNAseI (final concentration 1.4 μg/ml) and PMSF (final concentration 0.1 mM) were added. The sonicated sample was centrifuged and the supernatant was filtered (0.45 μm), before protein purification by nickel affinity chromatography using a HisTrap HP 5 ml column (GE Healthcare Life Sciences, Uppsala, Sweden) connected to an Äkta pure system (GE Healthcare Life Sciences, Uppsala, Sweden). A stepwise imidazole gradient ending at 500 mM imidazole was used to elute bound protein. After checking the presence and purity of the protein by SDS-PAGE, relevant fractions were pooled and the protein solution was concentrated, with concomitant buffer exchange to 20 mM Tris-HCl, 100 mM NaCl, pH 8.0, using Amicon Ultra-15 centrifugal filters with 10 000 NMWL (Merck Millipore, Cork, Ireland). The protein concentration was measured with the Bradford micro assay (Bio-Rad, CA, USA).

### 2.3 Structure determination

The protein solution (10 mg/ml) was mixed (1:1) with the crystallization solution (100 mM MES pH6.5 15–18% PEG 3350) for a final drop size of 4 μl. Crystallization was done in 24 well hanging drop plates. Rod shaped crystals appeared within 1–2 days at room temperature. For the cocrystallization experiments the protein solution (10 mg/ml) was treated with 1 mM EDTA (to prevent the catalysis) prior to the addition of (GlcNAc)_4_. Crystals were cryo-protected in the crystallization solution modified to include 30% ethylene glycol and flash cooled in liquid nitrogen.

X-ray diffraction data were collected at the European Synchrotron Radiation Facility ESRF Grenoble, France (collection statistics are summarized in Table 1). The images were integrated using the XDS [21] and XDSapp [22] software. The structures were solved by molecular replacement with Phaser [23] using the structure of *Sp*PgdA, a peptidoglycan deacetylase from *Streptococcus pneumoniae* (PDB id: 2C1G; [24]) as search model for 5LFZ and, subsequently, using 5LFZ as search model for 5LGC. The structures were refined by iterative cycles of PHENIX [25] and the CCP4 program REFMAC5 [26, 27] followed by the manual refitting of residues and ligands into the electron-density between the refinement cycles and placement of water molecules using Coot v.0.7.2 [28]. PRODRG [29] was used to generate the cif file for chitobiose.

**Table 1:** Crystallographic data and model statistics for the two structures.

Dataset	*Ar*CE4A-Ni^2+^	*Ar*CE4A-(GlcNAc)_2_
PDB code	5LFZ	5LCG
**Data collection**		
Source	ESRF ID29	ESRF ID29
Detector	Dectris Pilatis 6M	Dectris Pilatis 6M
Wavelength	0.97625	0.97625
No. of frames	1800	1350
Oscillation range per frame	0.1	0.1
**Diffraction data**		
Space group	P 2_1_ 2_1_ 2_1_	P 2_1_ 2_1_ 2_1_
Unit cell parameters	a = 39.09 Å b = 56.77 Å c = 76.86 Å	a = 40.49 Å b = 56.41Å c = 82.42 Å
No of measurements	155223	50017
Unique reflections	46454	11021
Resolution range	34.84–1.56	36.3–2.09
Completeness	99.7 (95.7)	93.9 (71.3)
Observed R factor (%)	4.1 (111)	5.2 (29.6)
R-meas	4.9 (133)	5.8 (33.9)
Rpim	2 (65.0)	2.4 (17.2)
Average I/sigmaCC(1/2)	12.82 (0.95) 99.9* (44.6*)	17.6 (3.7) 99.9* (93.6*)
**Refinement**		
Resolution limits	34.84–1.56	36.3–2.09
Free reflections	8.03%	5.15%
No. of protein atoms	1602	1558
No. of heterogen atoms	1	29
No. of waters	107	40
R factor (overall/free) (%)	0.178, 0.205	0.185, 0.223
Wilson B factor	24.6	33.3
R.m.s.d		
Bonds	0.005	0.006
Angles	0.957	0.930
Ramachandran		
Favored (%)	98	98
Allowed (%)	2	2
Outliers	0	0

* Correlation significant at the 0.1% level.

### 2.4 Activity assays

Reaction mixtures for determination of enzyme activity contained 2 mM or 5 mg/ml substrate, 10 μM CoCl_2_ and 300 nM enzyme in 50 mM Tris-HCl, pH 8.0. Reaction mixtures were incubated at 37°C, using a thermomixer with shaking at 600 rpm. Reactions were quenched by adding acetonitrile to a final concentration of 50% (v/v). *N*-acetylglucosamine (GlcNAc) was purchased from Sigma- Aldrich (Steinheim, Germany), while acetylated oligomers [(GlcNAc)_2-6_] were purchased from MegaZyme (Bray, Ireland). Alpha-chitin extracted from *Pandalus borealis* was from Seagarden (Avaldsnes, Norway) and β-chitin extracted from squid pen was purchased from France Chitin (Batch 20140101, Orange, France). Aspen acetyl xylan and chitosan (F_A_ = 0.64) were a kind gifts from Bjørge Westereng and BioCHOS AS (Ås, Norway), respectively. Quantification of released acetate was done by ion chromatography using a Dionex ICS3000 system with suppressed conductivity detection and equipped with a Dionex IonPac AS11 organic acid column, using the following gradient: 0–8 min, 1 mM KOH; 8–9 min, from 1 to 60 mM KOH; 9–16 min, 60 mM KOH; 16–16.1 min, from 60 to 1 mM KOH; 16.1–22 min, 1 mM KOH. The flow rate was 0.375 ml/min. The amount of released acetate was quantified using acetic acid [glacial, anhydrous (Merck, Damstadt, Germany)] as standard. Operation of the Dionex ICS3000 system and processing of chromatograms were performed using the Chromeleon 7 software (Dionex Corp.).

### 2.5 AMAC labeling and sequencing of chito-oligomers

Products generated by the deacetylase from (GlcNAc)_5_ were labeled with 2-aminoacridone (AMAC) (Sigma- Aldrich, Steinheim, Germany) as previously described by Bahrke et al. [30] and labeled products were purified using a C18 column (Starata C18E, Phenomenex, CA, US) as described by Morelle et al. [31], with one deviation: instead of lyophilizing the labeled samples, the reaction products were dried by vacuum centrifugation. The labeled products were re-dissolved in 50 μl 50% MeOH and analyzed using a LTQ-Velos Pro ion trap mass spectrometer (Thermo Scientific, Bremen, Germany) connected to an Ultimate 3000 RS HPLC (Dionex, CA, USA). This setup was used for direct injection without a column. The pump delivered 200 μl/min of 0.03 μM formic acid in 70% acetonitrile and data was acquired for 24 seconds after injection. For the MS, the capillary voltage was set to 3.5 kV and the scan range was *m/z* 150–2000 using two micro scans. The automatic gain control was set to 10,000 charges and a maximum injection time of 20 milliseconds. For fragmentation of desired precursor masses by MS2, the normalized collision energy was set to 37 and three micro scans were used. The data were recorded with Xcalibur version 2.2.

## 3. Results and discussion

### 3.1 Selection of candidate CDAs from metagenome data

Deacetylases in CAZy family CE4 contain five conserved motifs containing residues that are important for the catalytic activity [24, 32]: motif 1, T(F/Y)DD; motif 2, H(S/T)xxH; motif 3, R(P/x)PY; motif 4, DxxD(W/Y); motif 5, LxH. The second aspartate in motif 1 coordinates a metal ion, preferably Co^2+^ [24, 33], together with two histidines in motif 2. The first aspartate in motif 1 is believed to act as a base during catalysis, activating a water molecule to carry out a nucleophilic attack on the carbon in the scissile C-N bond. The histidine in motif 5, thought to be protonated, could promote C-N breaking by acting as an acid protonating the leaving amino-sugar. The backbone of motif 3, in particular of the tyrosine, is involved in stabilizing the oxyanion intermediate that is formed during catalysis [16, 24, 34]. Motif 3 and 4 each form one side of a shallow active site groove (Blair et al., 2005). Notably, proteins may receive a CE4 annotation without possessing all these five motifs and such CE4 enzymes are not likely to be active [35].

The initial search of the annotated bacterial genomes and metagenomes yielded 64 protein sequences (annotated as EC 3.5.1.41), 48 of which belonged to CAZy family CE4. Each sequence was manually inspected to check for the presence of all five sequence motifs, leaving 24 proteins. The genes for 8 of these 24 proteins did not seem complete, leaving 16 candidates. Considering that chitin would occur extracellularly, the next filter applied was the presence of a clear signal peptide, as predicted by SignalP 4.1 [36]. This filtering step left 5 candidates. At this point, probable multi-domain proteins (4 candidates) were excluded to increase the chances of successful expression. This left one candidate protein, for which a structural model was built using Swiss-Model [37–39] to verify for potential anomalies in or near the catalytic center. This novel CDA is the subject of the remaining part of this report. It is interesting to note that, after using this rather straightforward approach, 48 CE4 sequences only yielded one candidate CDA. Obviously, the discarded CE4s, without signal peptide and/or containing multi-domain proteins, could include active CDAs.

The selected CDA is 246 amino acids long, with a predicted signal peptide running from amino acid number 1 to 31. The protein originates from the Gram-positive bacterium *Arthrobacter* sp. AW19M34-1, which was isolated from a Tunicate located at 77 meters depth in Vestfjorden, Norway. Tunicates secrete a chitinous perithrophic membrane [40, 41] and *Arthrobacter* species are known for their ability to grow on chitin and for secretion of chitinases [42]. In line with commonly used nomenclature for CAZymes the CDA was named *Ar*CE4A. The gene sequence has been deposited in the European Nucleotide Archive under Accession number LT630322 (http://www.ebi.ac.uk/ena/data/view/LT630322).

### 3.2 Structure determination

**Fig 1:**
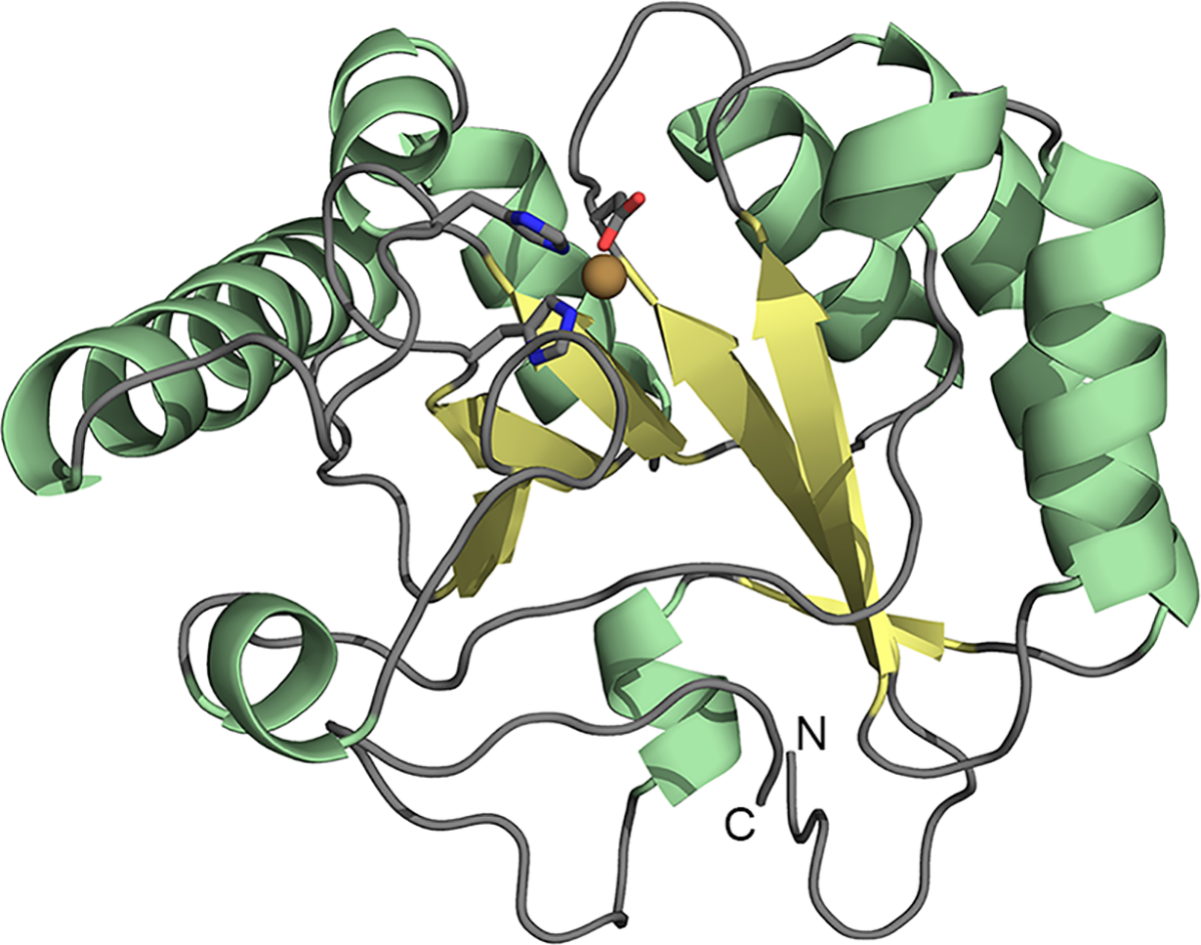
Cartoon representation of the ArCE4A showing the disrupted (β/α)8 barrel topology. The N- and C-terminus of the protein are marked and the metal ion in the active site is shown as a brown sphere, with the metal coordinating triad in sticks.

**Fig 2:**
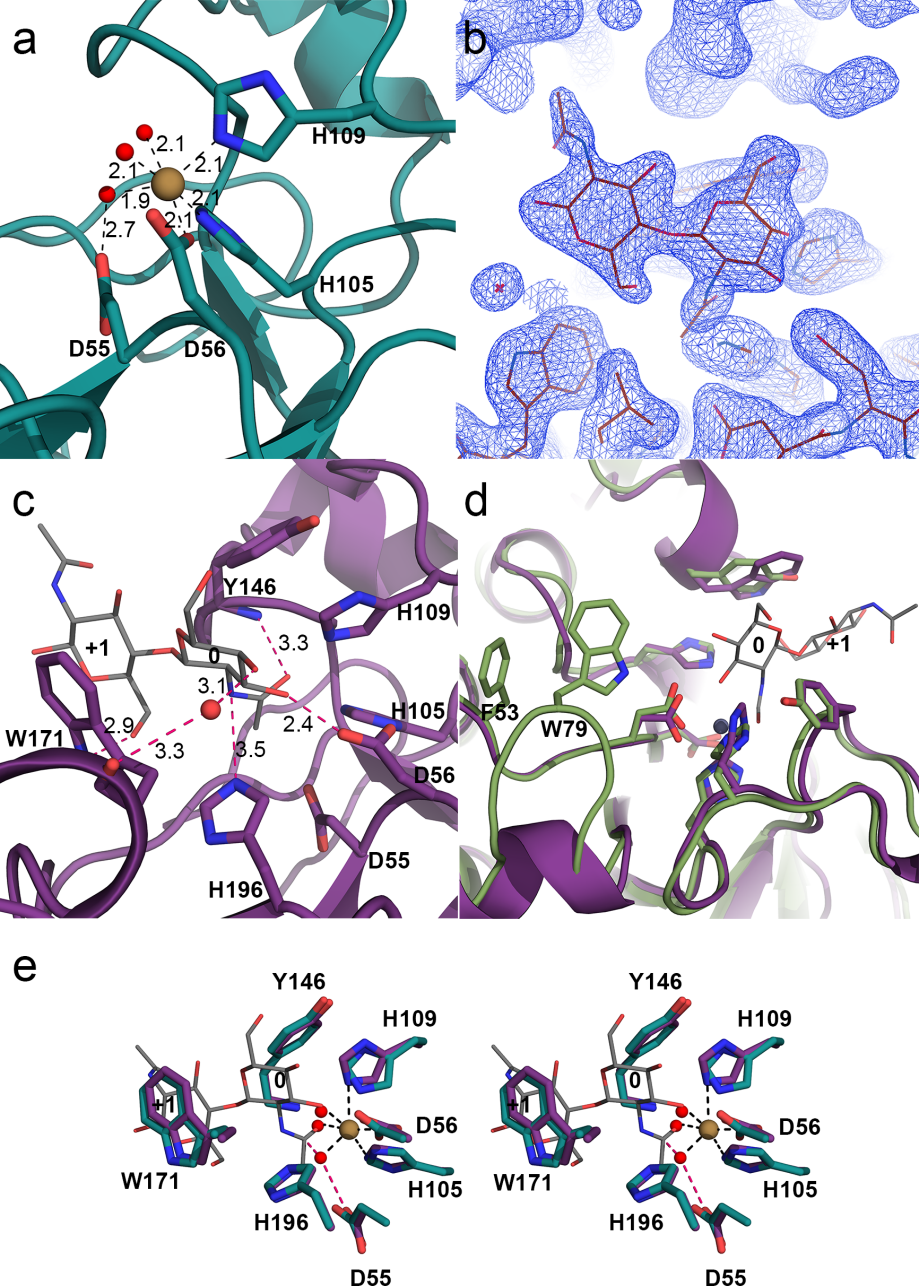
Structure of *Ar*CE4A determined by X-ray crystallography. (a) The His-His-Asp metal binding triad and the catalytic base (in sticks, PDB id: 5LFZ) with the Ni^2+^ ion as brown sphere. The Ni^2+^ ion shows octahedral coordination involving three amino acids and three water molecules (red spheres); interactions are shown as black dashed lines with distances in Å. The water molecule interacting with Asp55 is proposed to act as a nucleophile attacking the carbonyl carbon in the acetyl group. (b) Electron density map of the (GlcNAc)_2_ ligand. This illustrates the lack of electron density for the remainder of the tetramer used in the co-crystallization. (c) *Ar*CE4A in complex with (GlcNAc)_2_ (PDB id: 5LCG) showing active site with the ligand bound in subsites 0 and +1 (grey carbons). Residues involved in substrate binding and catalysis are shown as sticks (purple carbons). Interactions between the protein and the substrate are shown as dashed lines in pink with distances in Å. (d) Superposition of *Ar*CE4A (purple carbons) and *Cl*CDA (green carbons; PDB id: 2IW0 [34]), showing the extra loop containing Trp79 and nearby Phe53 (in sticks) in *Cl*CDA in what could be subsite -2. Subsites occupied by the ligand are labeled 0 and +1. (e) Cross-eyed stereo view of a superposition of the two structures (5LFZ in teal, 5LCG in purple) showing the active site cleft, and how the Ni^2+^ ion (brown sphere) and the three water molecules (red spheres) in 5LFZ are located relative to (GlcNAc)_2_ in 5LCG. Interactions involving the Ni^2+^ ion are shown as dashed black lines. Interactions between the proposed nucleophilic water and Asp55 and the carbonyl carbon in the acetyl group are shown as pink dashed lines.

Two structures of *Ar*CE4A were obtained by x-ray crystallography, one with (PDB id: 5LGC) and one without (PDB id: 5LFZ) a (GlcNAc)_2_ ligand, at 2.1 Å and 1.6 Å resolution, respectively (Table 1). The protein has a somewhat deformed (β/α)_8_ barrel topology (Fig 1) that is characteristic for CE4 proteins [15, 16, 24, 33, 34]. The structure of *Ar*CE4A without (GlcNAc)_2_ comprises residues 42–241, meaning that no structural information was obtained for ten N-terminal residues (32–41) and five C-terminal residues (242–246). Note that both the N- and the C-terminus are located on the opposite side of the protein, relative to the catalytic center (Fig 1). The structure contains a Ni^2+^ ion coordinated by Asp56, His105 and His109 (Fig 2A), which comprise the metal binding triad that is conserved in CE4 proteins. The Ni^2+^ most likely originates from the protein purification by nickel affinity chromatography. The Ni^2+^ ion is in an octahedral arrangement, involving three water ligands and the metal binding Asp-His-His triad. It has been proposed that one of these water molecules, coordinated by Asp55, is the catalytic water acting as a nucleophile during catalysis [24].

The structure with a bound ligand covers amino acids 41 to 239 and the ligand density (Fig 2B) was refined as a GlcNAc dimer, occupying subsite 0 and +1 (Fig 2C). From the four sugars of the (GlcNAc)_4_ that was used in the co-crystallization experiments, only two could be modeled into the electron density. Apparently, the other two sugars are not stabilized by any protein-substrate interactions and adopt multiple orientations/conformations that cannot be resolved in the electron density map at this resolution. Fig 2C shows that Trp171 in motif 4 stacks with the sugar bound in subsite +1 forming one side of a shallow substrate-binding groove. Binding of the sugar in the +1 subsite seems to be dominated by this stacking interaction, whereas the acetyl group of this sugar is not involved in interactions with the enzyme (Fig 2C). The sugar bound in subsite 0 has multiple interactions with the enzyme. The hydroxyl-group at C3 makes a hydrogen bond with Asp56 (Fig 2C and 2E), while the hydroxyl-group at C4 of the sugar bound in subsite 0 seems to have an indirect interaction with the backbone carbonyl of Trp171 through a water molecule (Fig 2C). Based on the superposition of the two structures it is likely that the hydroxyl-group at C3 also interacts with the metal ion (Fig 2E). The backbone amide of Tyr146, thought to stabilize the oxyanion intermediate by interacting with the oxygen atom of the acetyl group is located at 3.3 Å of this oxygen (Fig 2C). The Nε nitrogen of His196 in motif 5, thought to facilitate departure of the sugar, is located at 3.5 Å from the nitrogen atom in the acetamido group (Fig 2C), a distance not unlike the distances proposed in previous docking studies (3.7 Å; [24, 34]). Asp55 in motif 1, expected to activate the nucleophilic water is not making any direct interactions with the sugar in subsite 0. No water molecules could be refined in the active site cleft in the structure with the (GlcNAc)_2_ ligand, probably due to the lack of a metal ion. Superposition of the two *Ar*CE4A structures (Fig 2E) reveals that the water molecule coordinated by Asp55 in the substrate-free structure (Fig 2A) indeed has a position that could allow it acting as a nucleophile during catalysis. The other two water molecules, which coordinate the metal ion in the substrate-free enzyme (Fig 2A), occupy the same position as the oxygens of the acetyl group and the hydroxyl on carbon 3 in of the sugar bound in subsite 0. It is worth noting that the superposition (Fig 2E) shows little difference in the conformation of the above-mentioned amino acids.

**Fig 3:**
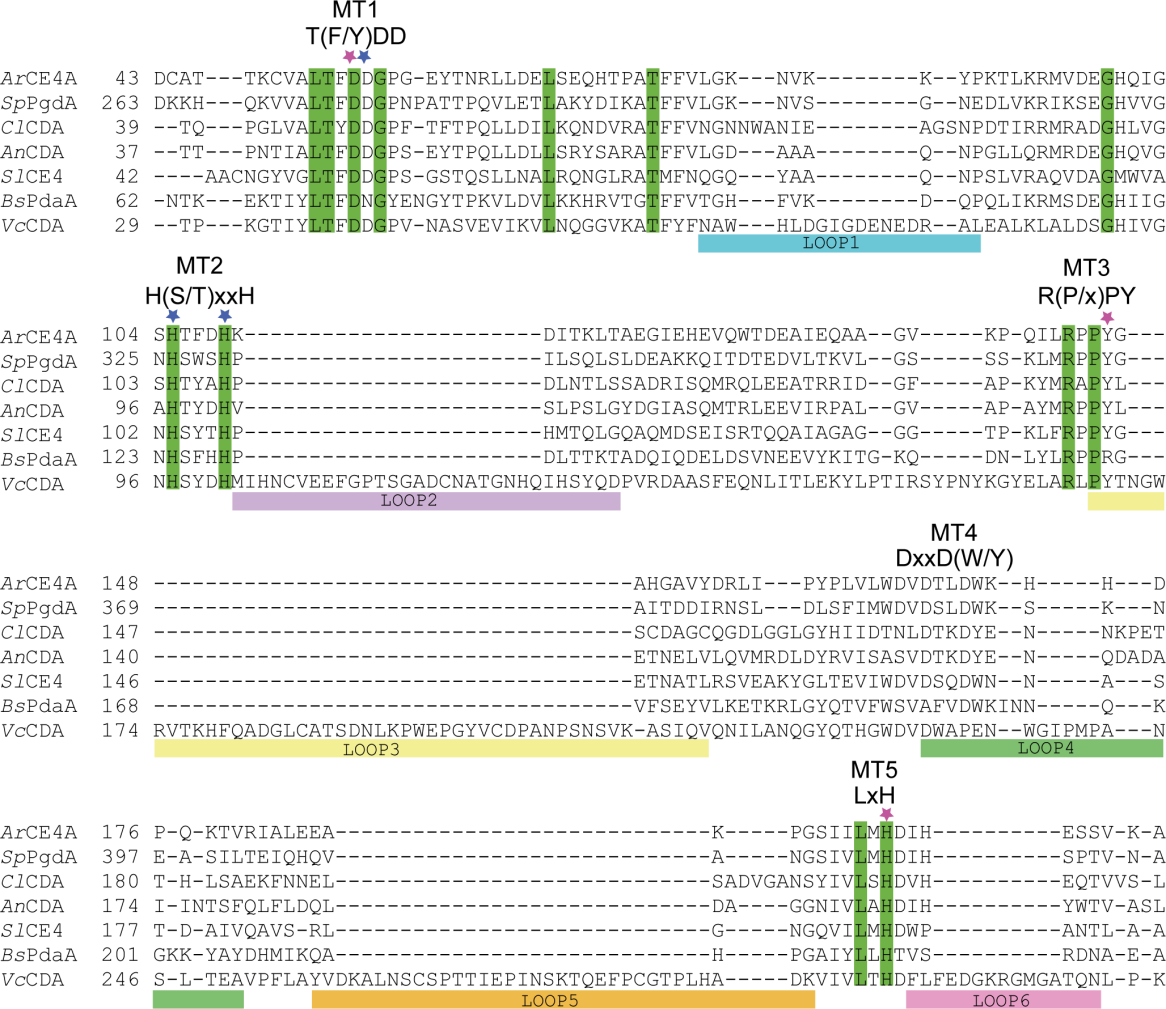
Structure-based sequence alignment of CE4 deacetylases. The structure-based sequence alignment was obtained using PyMod 1.0 [43]. Fully conserved residues are shown on a green background. The asterisks indicate residues involved in metal binding (blue) and in catalysis (pink). MT1-5 indicate the five conserved motifs in CE4 deacetylases. Colored horizontal bars indicate the different loops described by Andrés et al. [16]. The deacetylases included in the alignment are: *Sp*PgdA, PDB id 2C1G [24]; *Cl*CDA, PDB id 2IW0 [34]; *An*CDA, PDB id 2Y8U [15]; *Sl*CE4, PDB id 2CC0 [33]; *Bs*PdaA, PDB id 1W17 [44]; *Vc*CDA, PDB id 4NY2 [16]. For clarity, the alignment only shows the sequence area of the five motifs and the loops. Sequence numbering is based on the primary gene product, including the signal peptide for the proteins harboring a signal peptide.

A structure based sequence alignment with other known deacetylases (Fig 3) shows that there is high sequence similarity in the conserved motifs that are characteristic for deacetylases in family CE4. However, there is some variation, which could correlate with differences in substrate specificity, which are not all mapped yet, but are known to exist and be considerable. For example, *Bs*PdaA is an *N*-acetylmuramic acid deacetylase with no activity against CHOS [7], whereas *Vc*CDA only deacetylates CHOS on the sugar next to the non-reducing end. The structure of *Vc*CDA so far was the only available structure of a CE4 CDA in complex with its true substrate [16]. As shown in Fig 3, *Vc*CDA is special in that it contains several long insertions, which are loops that cover the active site and tailor this enzyme’s ability to interact with its substrate [16]. *Ar*CE4A and other CE4s proteins acting on CHOS have active sites that are more open. Based on biochemical data, Hekmat et al. (2003) proposed that *Cl*CDA, having an open active site similar to *Ar*CE4A, has four subsites, -2, -1, 0, and +1 [13]. The structure of *Cl*CDA was solved by Blair et al. [34] and based on *in silico* docking of (GlcNAc)_3_ they concluded that the sugar in subsite -1 has no interactions with the protein. Blair et al. further pointed out that a tryptophan (Trp79) located in an insertion in loop 1 that is absent in *Ar*CE4A (Figs 2D and 3) could create a -2 subsite [34]. A phenylalanine (Phe53, Fig 2D) located near the flexible loop with Trp79 could possibly also be involved in substrate binding in subsite -2 of *Cl*CDA. *Ar*CE4A is more open in the potential subsite -2 region (Fig 2D) without any obvious residues to make interactions with a bound sugar. Interestingly, while the protein was co-crystallized with (GlcNAc)_4_ only two GlcNAc units were observed. This suggests high flexibility of the rest of the ligand, which is in line with the notion that *Ar*CE4A has only two clear subsites, 0 and +1. Another noteworthy difference is the tyrosine in *Cl*CDA (Tyr173) in stead of a tryptophan in *Ar*CE4A (Trp171) in motif 4 [DxxD(W/Y), Fig 2D]. Of the 54 CE4 proteins listed in CAZy as characterized only *Cl*CDA [34] and *An*CDA [15] have a tyrosine in motif 4.

### 3.3 Enzymatic activity and substrate specificity

Functional features of *Ar*CE4A were investigated by testing the enzyme’s activity against different substrates and by sequence analysis of generated products. Table 2 shows the deacetylating activity of *Ar*CE4A for different substrates. For CHOS substrates, the apparent rate constant increased with increasing DP up to (GlcNAc)_5_, for which *Ar*CE4A has a higher apparent rate against (0.18 s^-1^) compared to (GlcNAc)_6_ (0.07 s^-1^). A similar pattern of activity against CHOS was observed for *An*CDA [15]. *Ar*CE4A did not deacetylate GlcNAc, and the activity against (GlcNAc)_2_ was very low. Next to CHOS, *Ar*CE4A deacetylates chitosan, chitin and acetyl xylan (Table 2).

**Table 2:** Activity of *Ar*CE4A against different substrates.

Substrate	Substrate concentration	ASAR^a^ (μM)	Average acetic acid release (μM)	CV%	Deacetylation degree (%)	Acetic acid released (nmol/min)	Apparent rate constant (s^-1^)
GlcNAc	2 mM	2000	0.0	0.0	0.000	0.00	0.00
(GlcNAc)_2_	2 mM	4000	0.1	14.5	0.003	0.00	0.00
(GlcNAc)_3_	2 mM	6000	11.9	2.6	0.20	0.04	0.02
(GlcNAc)_4_	2 mM	8000	39.8	2.8	0.50	0.13	0.07
(GlcNAc)_5_	2 mM	10000	95.8	7.7	0.96	0.32	0.18
(GlcNAc)_6_	2 mM	12000	39.4	0.2	0.33	0.13	0.07
Chitosan^b^	5 mg/ml	16000	85.4	1.4	0.53	0.28	0.16
α-chitin	5 mg/ml	24600^d^	0.8	6.4	0.003	0.00	0.00
β-chitin	5 mg/ml	24600^d^	1.4	1.9	0.006	0.001	0.00
Acetyl xylan^c^	5 mg/ml	9000	1696.7	3.1	18.9	5.66	3.14

The substrate was incubated with 300 nM *Ar*CE4A for 30 min at 37°C, and released acetic acid was measured by ion exchange HPLC.

^a^ASAR: amount of substrate expressed as the concentration of acetyl groups.

^b^The degree of acetylation was 64%.

^c^MW_avg_ = 2800, degree of acetylation roughly estimated to be around 50% by MALDI-TOF.

^d^Assuming one acetylation per sugar unit.

It is well known that CE4 enzymes tend to have broad substrate specificities. For example, enzymes classified as peptidoglycan deacetylases can deacetylate chito-oligomers [24, 32]. Likewise, CE4 enzymes known as acetylxylan esterases can deacetylate chitosan and CHOS [32, 45]. However, comparative information on rates is scarce. A recently described putative fungal CDA (*An*CDA) showed in general higher rates for various substrates [15], compared to *Ar*CE4A. Both *An*CDA and *Ar*CE4A are clearly most active towards acetylxylan and should thus perhaps, based on the available data, be classified as acetylxylan esterases [33, 45]. A further quantitative comparison of the activity of known CDAs towards chitinous substrates and acetylated plant polysaccharides such as acetylxylan would be of interest and could perhaps yield more insight into the true biological function of these enzymes.

**Fig 4:**
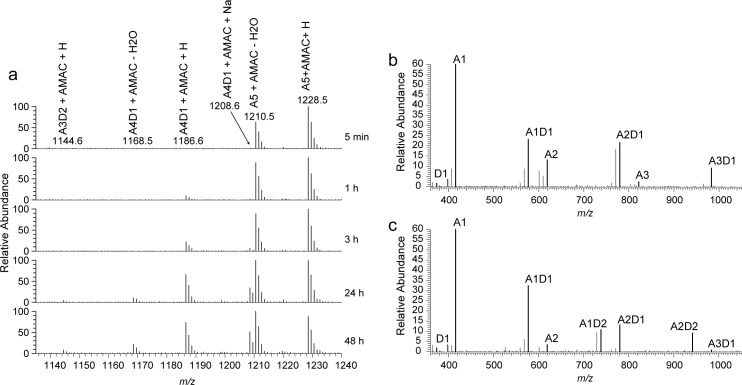
Mass spectrometric analysis of products generated from (GlcNAc)_5_. Reaction products generated upon treating (GlcNAc)_5_ with *Ar*CE4A were labeled with AMAC and analyzed by MS. (a) MS1 spectra of AMAC labeled reaction products at different reaction time points, showing appearance of mono- and di-deacetylated products. (b) Representative MS2 spectrum for the A4D1 peak at *m/z* 1186 from MS1 spectra (1h reaction time). (c) Representative MS2 spectrum for the A3D2 peak at *m/z* 1144 from MS1 spectra (24h reaction time). Bold signals in (b) and (c) correspond to the mass of the indicated CHOS plus AMAC and hydrogen. Reaction mixtures contained 2 mM A5 and 300 nM *Ar*CE4A and were incubated at 37°C.

Of the CHOS tested, *Ar*CE4A showed highest activity against (GlcNAc)_5_, and, therefore, this substrate was used for investigation of the position of deacetylation. The reducing ends of reaction products were labeled with AMAC and the resulting samples were analyzed using mass spectroscopy. MS1 spectra of AMAC-labeled products obtained at different reaction times (Fig 4A) show the initial appearance of mono-deacetylated products (*m/z* 1186.6) and the subsequent appearance of products with two deacetylations (*m/z* 1144.6) after 24 hours. The peaks for mono- and di-deacetylated products were isolated and subjected to fractionation in MS2 experiments (Fig 4B and 4C). Although a signal corresponding to GlcN-AMAC (*m/z* 374) is visible, a signal at *m/z* 416, corresponding to GlcNAc-AMAC, dominates in the MS2 spectra, indicating that the reducing end was hardly deacetylated. The MS2 spectrum for the mono-deacetylated product (Fig 4B) shows no signal that would indicate deacetylation of the non-reducing end (i.e. no A4-AMAC signal), indicating that the non-reducing end is not preferred for deacetylation. This may seem contradictory to the binding mode of the (GlcNAc)_2_ ligand seen in the structure where the non-reducing end is bound in subsite 0. It should be noted, however that the structure only shows part of the used substrate, (GlcNAc)_4_, and that it is thus not certain whether the chain “end” seen in the structure really is a chain end. The fact that no non-reducing end deacetylation is observed in Fig 4B may be taken to indicate that there must be some substrate affinity beyond subsites 0 and +1, in particular in what would be -1 and -2 subsites. Notably, the presence of a weak signal for A3D1 in Fig 4C, showing MS2 data for the double deacetylated product, shows that deacetylation of the non-reducing end did occur. The relative intensity of this signal is low, indicating that the non-reducing end is less preferred for deacetylation compared to the middle sugars of the pentamer.

The signals in Fig 4B show that the first deacetylation happens at all three internal positions. Although quantitative interpretation of the MS spectra is not very reliable, the data do seem to suggest that deacetylation near the reducing end is most frequent (suggested by the strong A1D1 signal). The products with two deacetylations seem to be dominated by deacetylation of the sugar next to the reducing end and of either of the two other internal sugars. The active site of *Cl*CDA bears resemblance to that of *Ar*CE4A (see Fig 2D) and the kinetics of this enzyme have been studied in detail. For *Cl*CDA acting on (GlcNAc)_4_, the first deacetylation is fast, while the subsequent deacetylations are slower [13]. It was also shown that *Cl*CDA deacetylates the reducing much more slowly than all other positions [13]. Our results indicate that, like in the case of *Cl*CDA, the reducing end is less preferred by *Ar*CE4A. This conclusion coincides with the structural data for the enzyme-substrate complex, showing a strong binding interaction in the +1 subsite. This suggests that *Ar*CE4A prefers a sugar bound in the +1 subsite for optimal activity, and thus will not be very active on reducing ends.

It should be noted that *Ar*CE4A showed very low activity against (GlcNAc)_2_ (Table 1), which suggests that occupation of more than two subsites, i.e. beyond subsite 0 and +1, is beneficial for activity. Currently available data do not allow a prediction of what additional interactions could benefit catalysis. Studies with *Vc*CDA, which, notably, has a very differently shaped catalytic center (see above), suggested that substrate-binding could lead to conformational changes, which in the case of *Ar*CE4A could lead to interactions that we cannot detect in the current data.

## 4. Concluding remarks

In this study, we present structural and functional data for *Ar*CE4A, including the first structural data for a complex between a low-specificity CE4 enzyme with an open active site and a substrate. While our motivation for this work was to develop enzymes for chitin processing, it is not certain that deacetylation of GlcNAc is the true biological function of *Ar*CE4A. If chitin were the natural substrate one would perhaps expect a higher activity against chitin, chitosan and CHOS compared to acetyl xylan (Table 2). A similar trend in substrate specificity was observed for *An*CDA, which is thought to be a fungal chitin deacetylase [15]. Interestingly, xylan is found in the cell wall of some marine algae [46], and it is therefore conceivable that certain marine bacteria may benefit from the ability to deacetylate this substrate. The broad substrate specificity observed for *Ar*CE4A and other CE4s [15, 45] is intriguing, and more comparable studies are needed to fully understand the substrate specificity.

The crystal structure of *Ar*CE4A in complex with (GlcNAc)_2_ provides a deeper understanding of how CE4 enzymes interact with their substrates, especially CE4s with an open active site, which are common in Nature. The structural data suggest that there are relatively few interactions between the substrate and the enzyme beyond subsites 0 and +1. The interaction in subsite +1 involves a tryptophan and is thus not very sugar specific, whereas more specific interactions in the form of hydrogen bonds occur in subsite 0. This interaction pattern is compatible with the observed broad specificity of the enzyme. It should be noted, however, that the activity of *Ar*CE4A against (GlcNAc)_2_ is low compared to other (longer) substrates, suggesting that unknown interactions, perhaps involving conformational changes, take place upon substrate binding (e.g. loop rearrangements [16]). Still, it is conceivable that a seemingly short and open substrate binding groove is an intentional feature of these enzymes in order to fit different substrates in the active site. Structural data for *Ar*CE4A in complex with longer substrate and different substrates would be of great interest and will be useful for better understanding the functionality of the CE4s. Such additional information may eventually also create possibilities for using these enzymes, or engineered variants thereof, to produce chitosans and CHOS with defined patterns of acetylation.
